# Biopsychosocial risk pathways to school violence in youth: a scoping review of stress reactivity, mental health outcomes, and prevention

**DOI:** 10.3389/frcha.2026.1895435

**Published:** 2026-07-14

**Authors:** Giuseppe Marano, Gianandrea Traversi, Osvaldo Mazza, Leila Farsakh, Daniele Napolitano, Alessio Simonetti, Antonio Maria D’Onofrio, Giovanni Camardese, Eleonora Gaetani, Marianna Mazza

**Affiliations:** 1 Department of Neuroscience, Head-Neck and Chest, Section of Psychiatry, Fondazione Policlinico Universitario Agostino Gemelli IRCCS, Rome, Italy; 2 Department of Neuroscience, Section of Psychiatry, Università Cattolica del Sacro Cuore, Rome, Italy; 3 Unit of Medical Genetics, Department of Laboratory Medicine, Ospedale Isola Tiberina-Gemelli Isola, Rome, Italy; 4 Spine Surgery Department, Bambino Gesù Children’s Hospital IRCCS, Rome, Italy; 5 SITRA and Scientific Direction-Fondazione Policlinico Gemelli IRCCS, Rome, Italy; 6 Department of Life Science, Health, and Health Professions, Link Campus University, Rome, Italy; 7 Department of Translational Medicine and Surgery, Fondazione Policlinico Universitario A. Gemelli IRCCS, Università Cattolica del Sacro Cuore, Rome, Italy; 8 Unit of Internal Medicine, Cristo Re Hospital, Rome, Italy

**Keywords:** adolescent mental health, bullying, cyberbullying, developmental psychopathology, emotional dysregulation, peer victimization, school violence, social determinants

## Abstract

School violence represents a major public health and educational concern with significant consequences for adolescents' psychological well-being, academic functioning, and social development. Increasing evidence indicates that violent behaviors in school environments arise from the complex interaction between individual vulnerabilities and broader social determinants. This scoping review aims to describe and discuss the available evidence on the risk factors, mental health outcomes, and prevention strategies associated with school violence. A comprehensive literature search was conducted across major databases, and studies were selected and charted according to predefined inclusion criteria. The evidence was analyzed following a developmental psychopathology framework. We identified and analyzed key individual risk factors, including emotional dysregulation, impulsivity, empathy deficits, trauma exposure, and psychiatric vulnerabilities such as attention-deficit/hyperactivity disorder, conduct disorder, and mood disorders. In addition, we mapped contextual determinants including family functioning, peer dynamics, school climate, and the growing role of digital environments, particularly cyberbullying. The review further categorized the psychological profiles of aggressors, victims, and bully-victims, highlighting the developmental fluidity of these roles. Attention was also given to biological and psychophysiological pathways linking school violence, trauma exposure, and adolescent mental health, including stress-system dysregulation, hypothalamic-pituitary-adrenal axis functioning, autonomic arousal, threat sensitivity, and neurodevelopmental vulnerability. Within a biopsychosocial framework, these mechanisms are discussed as potential mediators between adverse social experiences, emotional dysregulation, aggression, victimization, and subsequent internalizing or externalizing outcomes. We synthesized evidence on the mental health consequences associated with exposure to school violence, including depression, anxiety, post-traumatic stress symptoms, and suicidal ideation. Finally, evidence-based prevention and intervention strategies are discussed, emphasizing integrated approaches that combine school-based programs, family involvement, and collaboration with mental health services to support early identification and prevention. This review provides a biopsychosocial mapping of the current literature and highlights the need for future studies integrating psychosocial assessment with biological and psychophysiological measures of stress reactivity and neurodevelopmental vulnerability.

## 1 Introduction

School violence represents a major public health and educational concern worldwide, with significant consequences for adolescents’ psychological well-being, academic functioning, and social development. Violent behaviors in school settings include a broad range of actions such as physical aggression, verbal harassment, relational aggression, and bullying occurring both in traditional and digital contexts. Over the past decades, re-search has increasingly highlighted the widespread prevalence of school violence and its negative impact on students' mental health, academic performance, and long-term psychosocial adjustment (1).

Bullying, one of the most extensively studied forms of school violence, is typically defined as repeated aggressive behavior characterized by an imbalance of power between the perpetrator and the victim (2). Epidemiological studies indicate that a substantial pro-portion of adolescents are involved in bullying dynamics during their school years, either as perpetrators, victims, or bully-victims. Large-scale international studies suggest that approximately 20%–30% of school-aged youth report some form of involvement in bullying behaviors (3). Exposure to bullying and peer victimization has consistently been associated with a wide range of negative psychological outcomes, including depression, anxiety, self-harm behaviors, and suicidal ideation (1, 4).

From a theoretical perspective, the framework of developmental psychopathology provides an important lens through which to understand the emergence of aggressive and violent behaviors during childhood and adolescence. This perspective emphasizes how maladaptive developmental trajectories arise from the dynamic interaction between biological vulnerabilities, psychological traits, and environmental stressors across developmental stages (5, 6). Within this framework, school violence can be conceptualized not as an isolated behavioral problem but as the outcome of complex developmental processes shaped by multiple interacting risk and protective factors.

Several individual-level psychological vulnerabilities have been associated with aggressive and violent behaviors among adolescents. Emotional dysregulation, impulsivity, deficits in empathy, and exposure to traumatic or adverse childhood experiences may significantly increase the risk of aggressive conduct and peer conflict (7). Moreover, specific psychiatric conditions, including attention-deficit/hyperactivity disorder (ADHD), conduct disorder, and mood disorders, have been linked to an increased likelihood of involvement in bullying dynamics (8, 9).

Beyond individual vulnerabilities, social and environmental determinants also play a crucial role in shaping violent behaviors in school settings. Family functioning, parenting practices, peer relationships, and the broader school climate represent important contextual factors that may either exacerbate or mitigate aggressive behaviors among students (10, 11). In recent years, the rapid expansion of digital communication technologies has also introduced new forms of peer aggression, particularly cyberbullying, which may extend victimization beyond school boundaries and intensify its psychological impact (12, 13).

Importantly, the roles of aggressor and victim are not necessarily fixed categories. Longitudinal studies suggest that adolescents may shift between these roles over time, and some individuals may simultaneously experience both victimization and aggressive behavior, forming the group often referred to as bully-victims (14). This dynamic interaction underscores the importance of adopting multidimensional and developmental perspectives in order to better understand the complexity of school violence.

Given the growing recognition of the multifactorial determinants of school violence and its significant mental health implications, there is an increasing need for integrative models that combine psychological, social, and developmental perspectives. The present scoping review aims to synthesize and map current evidence on the psychological and social pathways underlying school violence from a developmental psychopathology perspective. Specifically, the review examines individual psychological vulnerabilities, social and environmental determinants, mental health consequences associated with bullying involvement, and evidence-based prevention strategies.

School violence may also be conceptualized as a repeated interpersonal stressor with potential biological consequences during sensitive periods of child and adolescent development. Experiences such as peer humiliation, intimidation, social exclusion, relational aggression, and cyberbullying may activate stress-response systems involved in neuroendocrine regulation, autonomic arousal, immune-inflammatory signaling, and threat processing. In this perspective, bullying is not only a social or behavioral phenomenon, but also a potential pathway through which chronic social threat becomes biologically embedded. Altered hypothalamic-pituitary-adrenal axis functioning, atypical cortisol secretion, sympathetic hyperarousal, reduced parasympathetic flexibility, heightened threat sensitivity, and inflammatory activation may contribute to the development of internalizing and externalizing outcomes among vulnerable youth (15–17). Therefore, a biologically informed biopsychosocial model is needed to clarify how school violence influences adolescent mental health and why similar experiences may lead to heterogeneous clinical trajectories, including depression, anxiety, post-traumatic stress symptoms, reactive aggression, and suicidal ideation.

The present scoping review aims to map current evidence on school violence and bullying involvement through a biologically informed biopsychosocial framework. Specifically, the review examines: psychological and psychiatric vulnerabilities associated with aggression, victimization, and bully-victim profiles; biological and psychophysiological mechanisms linking peer victimization to adolescent mental health, including HPA-axis functioning, cortisol regulation, autonomic arousal, stress reactivity, inflammatory pathways, and threat sensitivity; social and environmental determinants, including family functioning, peer dynamics, school climate, and digital environments; prevention and intervention strategies that may reduce both psychosocial risk and stress-related biological dysregulation. 

## 2 Materials and methods

This scoping review was conducted and reported in accordance with the Preferred Reporting Items for Systematic Reviews and Meta-Analyses extension for Scoping Reviews (PRISMA-ScR). The PRISMA-ScR approach was selected because the purpose of the review was to map the breadth, characteristics, and conceptual organization of a heterogeneous body of evidence rather than to estimate a pooled intervention effect. The completed PRISMA-ScR checklist is provided as Supplementary Material S1.

The review was structured around the following questions: (1) which individual psychological and psychiatric factors are associated with school violence, bullying perpetration, victimization, or bully-victim status among children and adolescents? (2) which biological and psychophysiological mechanisms may contribute to the association between school violence and mental health outcomes? (3) which family, peer, school, community, and digital-context factors increase or reduce the risk of school violence? and (4) which prevention and intervention pathways have been described in the literature?

The review process included identification of potentially relevant records, duplicate removal, title and abstract screening, full-text eligibility assessment, data charting, and qualitative evidence mapping. Because the review addressed evidence from multiple disciplines and included different study designs, no quantitative meta-analysis was planned.

The aim of this scoping review was to map and synthesize the available literature on school violence within a developmental psychopathology framework, with particular attention to individual psychological risk factors, social and environmental determinants, mental health outcomes, and prevention/intervention strategies. The scoping methodology was chosen because of the broad and heterogeneous nature of the topic and the objective of identifying key concepts, research trends, and evidence gaps across different study designs and disciplinary fields.

### 2.1 Information sources and search strategy

A comprehensive literature search was conducted in PubMed/MEDLINE, Scopus, and Web of Science Core Collection to identify relevant publications issued between January 1, 2000, and January 31, 2025. The reference lists of eligible articles and relevant reviews were also manually screened to identify additional publications. Search concepts were organized into four principal blocks: school violence and bullying; child and adolescent populations; psychological, psychiatric, social, and contextual risk factors; and biological or psychophysiological stress mechanisms. Controlled vocabulary, where available, was combined with free-text terms using the Boolean operators AND and OR.

The search included terms related to school violence and peer aggression, such as “school violence,” “bullying,” “peer victimization,” “adolescent aggression,” and “cyberbullying”; psychological and contextual factors, including “emotional dysregulation,” “impulsivity,” “self-esteem,” “substance use,” “family functioning,” “school climate,” “community disadvantage,” and “adolescent mental health”; and biological or psychophysiological mechanisms, including “stress reactivity,” “hypothalamic-pituitary-adrenal axis,” “cortisol,” “autonomic functioning,” “heart rate variability,” “physiological arousal,” “neuroendocrine,” “inflammation,” “allostatic load,” “trauma biology,” and “neurodevelopmental vulnerability.” The complete database-specific search strategies, including the Boolean operators, search fields, limits, final search dates, and numbers of records retrieved, are presented in Supplementary Table S1. The search strategies were adapted to the syntax and indexing characteristics of each database.

### 2.2 Eligibility criteria

Studies were eligible when they: (1) examined children or adolescents, predominantly within the age range of 6–18 years; (2) addressed school violence, traditional bullying, peer victimization, relational aggression, cyberbullying, or bully-victim involvement; and (3) reported evidence concerning psychological or psychiatric vulnerabilities, biological or psychophysiological mechanisms, family or peer determinants, school or community context, mental health outcomes, or prevention and intervention strategies. Empirical quantitative, qualitative, and mixed-method studies, as well as systematic reviews, meta-analyses, and theoretically relevant papers, were considered because of the broad evidence-mapping objective of the review.

Publications were excluded when they: focused exclusively on adult or non-school populations; addressed violence without a clear school, peer, or youth-related component; did not report outcomes relevant to the review questions; were not available in English; or consisted of editorials, letters, conference abstracts, or publications containing insufficient information for evidence mapping.

### 2.3 Study selection

Following duplicate removal, two reviewers (G.M. and M.M) independently screened titles and abstracts against the predefined eligibility criteria. Records considered potentially relevant by either reviewer were retained for full-text assessment. The same reviewers independently evaluated the full texts and documented the principal reason for exclusion. Disagreements were resolved through discussion and, when consensus could not be reached, consultation with a third reviewer (G.T). The reviewers were not blinded to journal titles, study authors, or institutional affiliations, as blinding is not routinely required in scoping-review screening.

The data-base search identified 1,248 records, and an additional 37 records were identified through manual searches of reference lists and other sources, for a total of 1,285 records. After removal of 312 duplicates, 973 records remained for title and abstract screening. Of these, 845 records were excluded as not relevant to the review topic. A total of 128 full-text articles were sought for retrieval; 9 reports were not retrieved. Thus, 119 full-text articles were assessed for eligibility. Of these, 73 were excluded for the following reasons: not specifically focused on school violence or peer aggression (*n* = 24), adult or non-school population (*n* = 15), non-English language (*n* = 8), no relevant mental health or prevention out-comes (*n* = 14), or editorial/commentary/insufficient data for evidence mapping (*n* = 12). Finally, 46 studies were included in the scoping review. The scoping review was not prospectively registered in an international database.

### 2.4 Data charting and qualitative evidence mapping

A standardized data-charting matrix was developed before the final evidence synthesis. For each included study, the following information was extracted, when available: first author and publication year; country; study design; sample size; participant age range; school level or setting; type of violence or bullying involvement; participant role as perpetrator, victim, or bully-victim; psychological or psychiatric variables; biological or psychophysiological measures; family, peer, school, community, and digital-context variables; mental health, behavioral, academic, or social outcomes; prevention or intervention characteristics; and principal findings relevant to the review questions.

Data charting was performed by G.M. A second author (M.M.) independently verified all entries. Discrepancies were resolved through discussion and reference to the original publication. Because the purpose was evidence mapping rather than calculation of a pooled effect estimate, the synthesis was qualitative and thematic.

The qualitative mapping followed a deductive-inductive process. An initial coding framework was derived from the review questions and the biopsychosocial model, comprising: (1) individual psychological and psychiatric vulnerabilities; (2) biological and psychophysiological pathways; (3) family and peer determinants; (4) school, community, and digital contexts; (5) mental health, behavioral, academic, and social consequences; and (6) prevention and intervention strategies. During full-text charting, additional subthemes were added when recurrent constructs were identified. Studies could be assigned to more than one domain because the categories were considered interacting rather than mutually exclusive. The final thematic structure was reviewed by the author group to ensure conceptual consistency and was subsequently used to organize the narrative synthesis, tables, and figures. The complete standardized data-charting matrix used to organize the evidence is presented in Supplementary Table S2.

No formal risk-of-bias assessment was undertaken because the primary objective was to characterize the range and organization of the available evidence rather than determine a pooled estimate of effectiveness. This choice is consistent with the mapping purpose of a scoping review; however, the methodological limitations of the included literature were considered when interpreting the findings.

**Figure 1:**
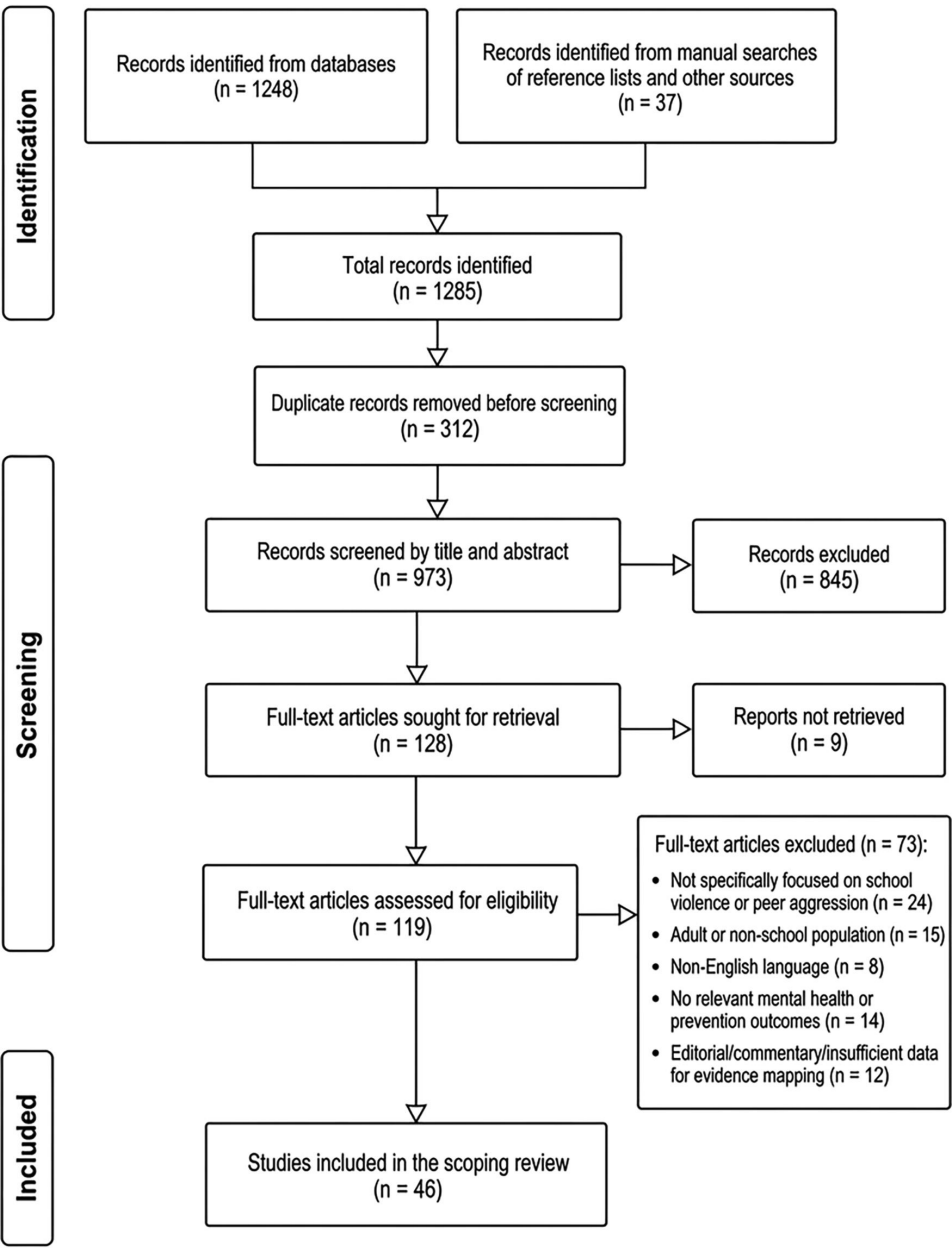
PRISMA flow diagram of the study selection process.

A PRISMA flow diagram summarizing the study selection process is presented in Figure 1.

**Figure 2:**
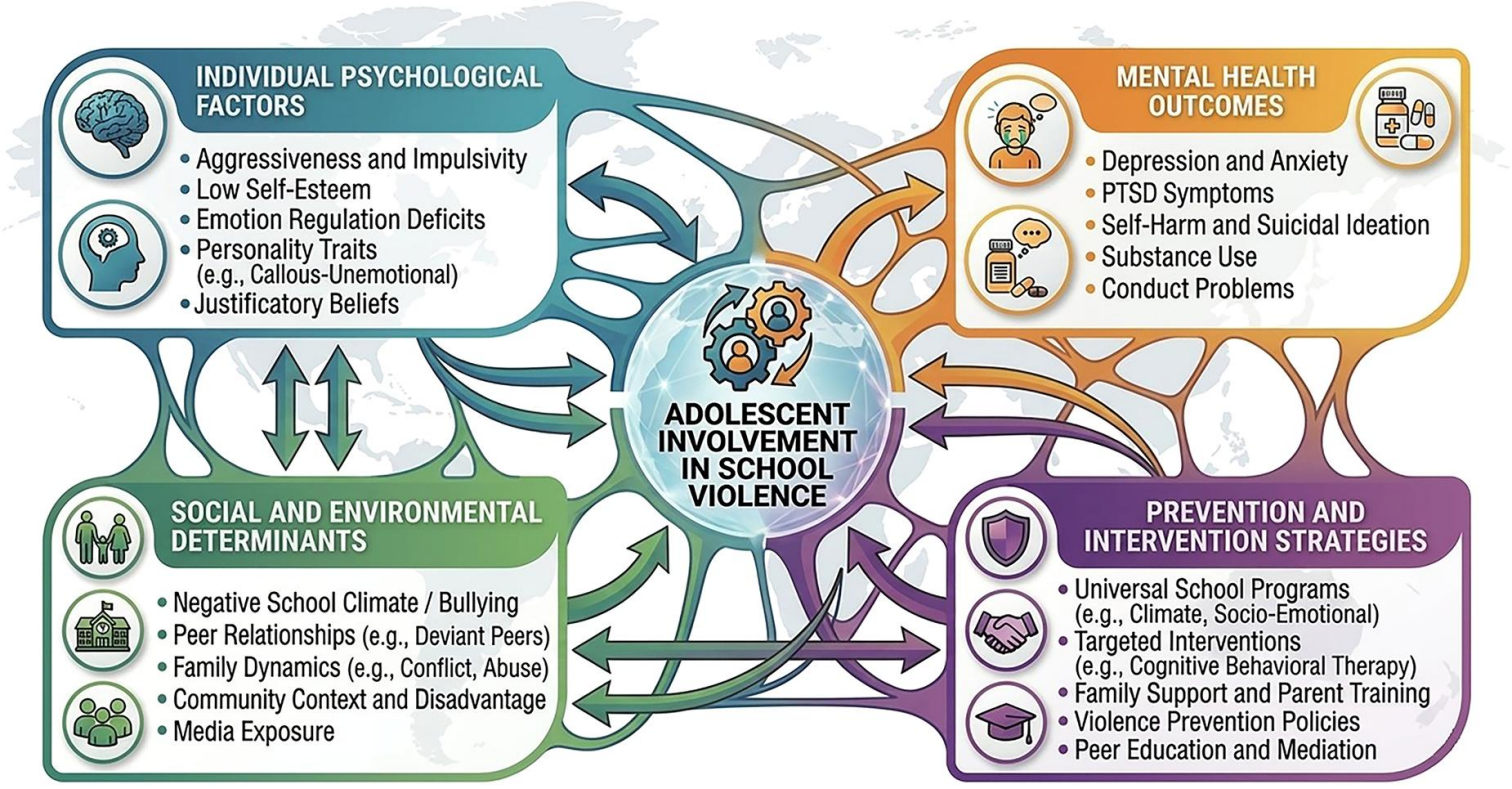
Conceptual framework illustrating the main domains addressed in this scoping review, including individual factors, social/environmental determinants, mental health outcomes, and prevention strategies. The four main domains addressed in this review, including individual psychological factors, social and environmental determinants, mental health outcomes, and prevention and intervention strategies, interact dynamically, with risk and protective factors at multiple levels influencing adolescents' involvement in school violence. This framework summarizes the evidence mapping approach used in the review and illustrates pathways that inform evidence-based prevention and intervention strategies.

All selected studies were charted and mapped according to the review's predefined domains, capturing individual psychological factors, social and environmental determinants, mental health outcomes, and prevention/intervention strategies. To visually represent the organization of these domains and their interconnections, a conceptual framework was developed (Figure 2). This framework illustrates how individual vulnerabilities, social and environmental factors, and contextual influences interact to shape adolescents' risk of involvement in school violence and how these domains inform prevention and intervention strategies. This scoping review provides a structured overview of the psycho-logical, social, and developmental determinants of school violence, supporting future re-search directions and informing evidence-based practice in educational and mental health contexts.

## 3 Individual psychological risk factors for school violence

Understanding the psychological determinants of aggressive and violent behaviors in school environments is essential for developing effective prevention and intervention strategies. From a developmental psychopathology perspective, school violence emerges from the interaction between individual vulnerabilities and environmental influences across childhood and adolescence (5, 18]). Several individual psychological characteristics have been consistently associated with an increased risk of involvement in bullying and other forms of school aggression, including emotional dysregulation, impulsivity, deficits in empathy, exposure to trauma, and the presence of specific psychiatric conditions.

### 3.1 Emotional dysregulation

Emotional regulation refers to the processes through which individuals monitor, evaluate, and modify emotional responses in order to achieve adaptive functioning (19). Difficulties in emotion regulation have been widely recognized as a key psychological vulnerability associated with aggressive behavior in children and adolescents. Youth who struggle to regulate negative emotions such as anger, frustration, and fear may be more likely to react impulsively or aggressively in interpersonal situations.

**Figure 3:**
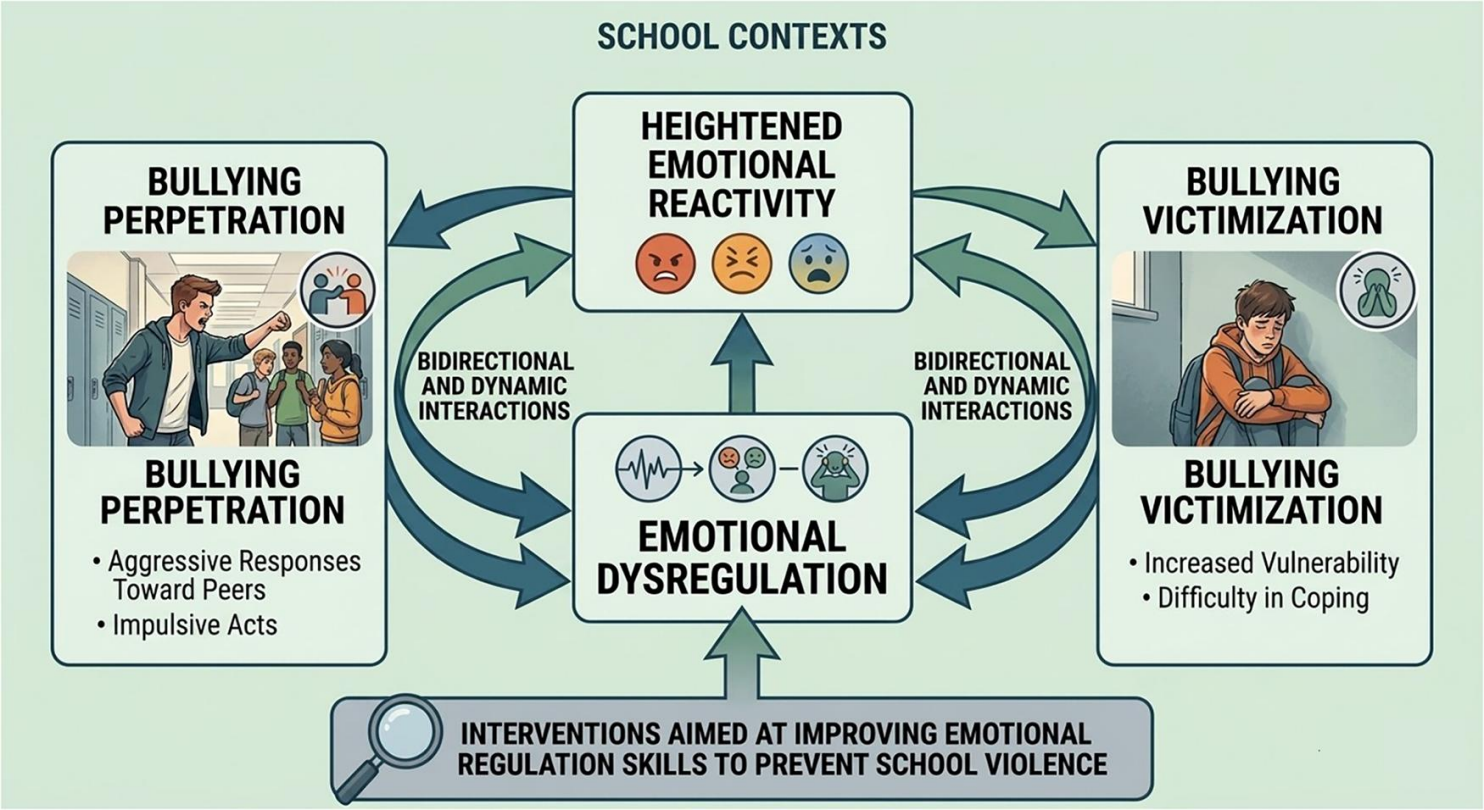
Conceptual pathway linking emotional dysregulation to school violence outcomes. Difficulties in regulating emotions such as anger, frustration, or fear increase adolescents' emotional reactivity, which may lead to aggressive responses toward peers (perpetration) and simultaneously increase vulnerability to peer victimization. The bidirectional and dynamic interactions between emotional dysregulation and social outcomes in school contexts are highlighted, emphasizing the importance of interventions aimed at improving emotional regulation skills to prevent school violence.

Empirical studies have demonstrated that deficits in emotional regulation are strongly associated with both bullying perpetration and victimization. Adolescents with poor emotional regulation skills often experience heightened emotional reactivity and reduced capacity to manage interpersonal conflicts effectively (5). These difficulties may contribute to maladaptive coping strategies, including aggressive responses toward peers. Figure 3 illustrates how emotional dysregulation can contribute to both perpetration and victimization in school contexts.

Furthermore, emotional dysregulation has also been linked to increased susceptibility to peer victimization, as emotionally reactive children may appear more vulnerable within social hierarchies (20). Emotional regulation skills develop gradually across childhood and adolescence and are shaped by both biological and environmental factors, including parenting practices, attachment relationships, and exposure to stress (21). Consequently, interventions aimed at improving emotional regulation abilities represent a key component of many school-based violence prevention programs.

### 3.2 Impulsivity and behavioral control

Impulsivity and deficits in behavioral inhibition represent another important psychological risk factor for aggressive behavior in school settings. Impulsivity refers to a tendency to act quickly without adequate consideration of potential consequences, often resulting in maladaptive behaviors and interpersonal conflicts.

Research evidence indicates that adolescents with high levels of impulsivity are more likely to engage in aggressive acts, including physical and verbal aggression toward peers. Poor inhibitory control may limit an individual's capacity to pause and evaluate social situations before reacting, thereby increasing the likelihood of hostile responses during interpersonal conflicts (22). Impulsivity has also been linked to difficulties in problem-solving and reduced ability to employ constructive conflict resolution strategies. Moreover, impulsivity frequently co-occurs with other psychological traits associated with aggression, such as sensation seeking, emotional instability, and deficits in executive functioning, which may further increase vulnerability to involvement in bullying dynamics.

**Figure 4:**
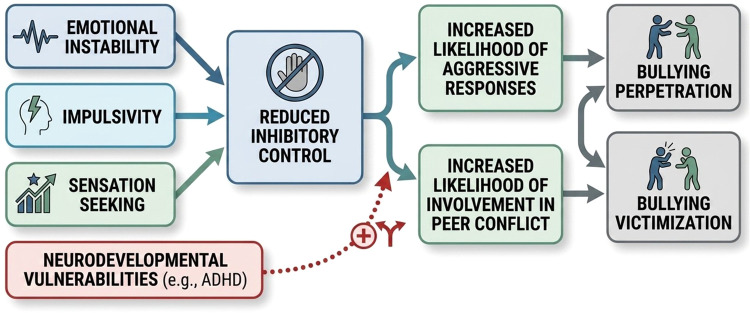
Impulsivity and deficits in behavioral control leading to aggression and peer conflict. Impulsivity, emotional instability, and sensation seeking reduce inhibitory control, increasing the likelihood of aggressive responses and involvement in peer conflict, while neurodevelopmental vulnerabilities, such as attention-deficit/hyperactivity disorder (ADHD), further amplify the risk for bullying perpetration and victimization.

Importantly, impulsivity is a central feature of several neurodevelopmental disorders, including ADHD, which has been consistently associated with elevated rates of peer conflict and aggressive behavior. Figure 4 shows how high impulsivity, emotional instability, and sensation seeking reduce behavioral control, increasing the likelihood of aggression and peer conflict.

### 3.3 Empathy deficits and moral disengagement

Empathy, defined as the ability to understand and share the emotional states of others, plays a critical role in regulating social behavior and inhibiting aggression. Low levels of empathy have been consistently associated with increased aggressive and antisocial behaviors among adolescents (7).

Adolescents with deficits in affective or cognitive empathy may have difficulty recognizing the emotional impact of their behavior on others, reducing natural inhibitory mechanisms against aggression. Empirical studies show that youth involved in bullying perpetration often exhibit lower levels of empathic concern compared with non-aggressive peers (23).

Closely related to empathy deficits is moral disengagement, a cognitive process through which individuals justify or rationalize harmful behavior toward others (24). Mechanisms such as victim blaming, minimization of harm, or diffusion of responsibility allow individuals to disengage from internal moral standards, legitimizing aggressive conduct. Moral disengagement has been identified as a significant predictor of bullying behavior in longitudinal studies (23).

Reduced empathy and increased moral disengagement contribute to the persistence of aggressive behaviors in peer relationships and facilitate the normalization of violence within certain social contexts.

### 3.4 Trauma and adverse childhood experiences

Exposure to early-life adversity represents another important psychological pathway linking developmental vulnerabilities to aggressive behaviors. Adverse childhood experiences (ACEs), including physical abuse, emotional neglect, household dysfunction, and exposure to domestic violence, have been systematically associated with elevated risk of behavioral problems and interpersonal aggression during adolescence (25).

Trauma exposure can disrupt emotional development, stress regulation systems, and social functioning. Children experiencing chronic stress or trauma often develop heightened threat sensitivity, difficulties in emotional regulation, and maladaptive coping strategies, which may increase the likelihood of aggressive responses in social interactions (26).

**Figure 5:**
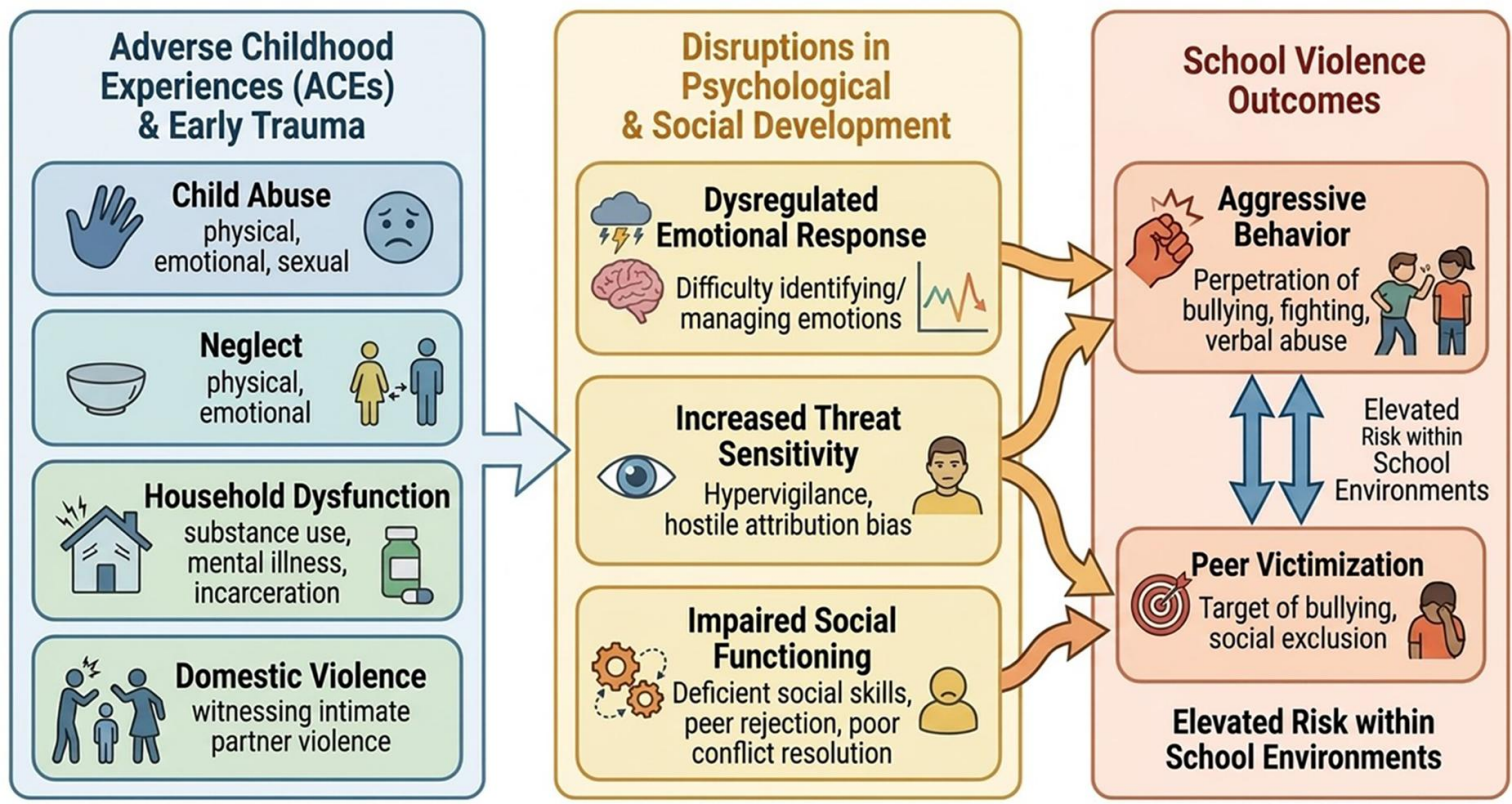
Pathways connecting trauma and adverse childhood experiences to school violence out-comes. Early-life adversity (abuse, neglect, household dysfunction, domestic violence) disrupts emotional regulation, increases threat sensitivity, and impairs social functioning, thereby elevating the risk of aggressive behavior and peer victimization in school environments.

Additionally, trauma-exposed youth may display increased hostility, mistrust, or social withdrawal, potentially contributing to cycles of aggression and victimization within school settings. At the same time, adolescents with histories of trauma may be particularly vulnerable to peer victimization due to heightened psychological vulnerability and social difficulties. Figure 5 depicts the pathways linking trauma and ACEs to heightened risk of aggressive behavior and victimization in school contexts.

### 3.5 Low self-esteem and substance use

Low self-esteem may function as both a vulnerability factor and a consequence of involvement in school violence. Adolescents with a persistently negative self-evaluation may experience greater sensitivity to rejection, reduced assertiveness, and difficulty mobilizing protective peer support, potentially increasing vulnerability to victimization. Recurrent humiliation, exclusion, and stigmatization may in turn erode perceived self-worth, creating a reciprocal pathway between victimization and low self-esteem (1, 4). Among perpetrators, apparent social dominance does not necessarily indicate stable self-worth; in some individuals, aggressive behavior may serve as a compensatory strategy for managing perceived inadequacy, social threat, or unstable self-evaluation. Low self-esteem should therefore be interpreted as a transdiagnostic construct that may precede, accompany, or follow bullying involvement rather than as a simple unidirectional cause.

Substance use represents another clinically relevant correlate of school violence. Alcohol, nicotine, cannabis, and other substance use may co-occur with bullying perpetration, victimization, and particularly bully-victim status. Several pathways may account for this association. Substance use may increase disinhibition, impair judgment, and intensify impulsive or retaliatory aggression. Conversely, victimized adolescents may use substances as a maladaptive coping strategy to reduce distress, social anxiety, intrusive memories, or feelings of exclusion. Both substance use and school violence may also reflect shared underlying vulnerabilities, including impulsivity, conduct problems, family adversity, deviant peer affiliation, weak school connectedness, and exposure to community violence (5). The association should therefore not be interpreted as proof that substance use directly causes school violence. Its presence nonetheless identifies a subgroup that may require integrated assessment of behavioral, emotional, family, and addiction-related risks.

### 3.6 Biological and psychophysiological mechanisms

Although school violence is often examined through psychological, relational, and con-textual lenses, a growing body of evidence suggests that biological and psychophysiological mechanisms may represent important pathways linking peer victimization, aggressive behavior, and adolescent mental health outcomes. From a developmental psychopathology perspective, bullying and school violence can be conceptualized as chronic interpersonal stressors occurring during sensitive periods of neurodevelopment. Repeated exposure to humiliation, social exclusion, intimidation, or perceived threat may activate bio-logical stress-response systems, including the hypothalamic-pituitary-adrenal (HPA) axis, the autonomic nervous system, immune-inflammatory pathways, and neural circuits involved in threat detection and emotional regulation (15–17).

One of the most widely investigated biological mechanisms in this field is HPA-axis functioning. Peer victimization and chronic social stress may influence cortisol secretion patterns, although findings across studies remain heterogeneous. In a systematic review, Kliewer et al. (17) showed that peer victimization has been associated with altered cortisol production in children and adolescents, suggesting that bullying-related stress may be-come biologically embedded through changes in neuroendocrine functioning. Some studies indicate heightened cortisol responses, whereas others suggest blunted cortisol reactivity, particularly after repeated or prolonged exposure to social threat (17). These apparently divergent patterns may reflect different developmental stages, timing of exposure, chronicity of victimization, and individual differences in stress-system adaptation. Within an allostatic load framework, repeated activation of the HPA axis may gradually reduce the organism's capacity to respond flexibly to new stressors, contributing to emotional instability, hypervigilance, sleep disruption, anxiety, depressive symptoms, and trauma-related responses (15, 16).

Empirical studies also support a link between bullying victimization, cortisol regulation, and cognitive-emotional outcomes. Vaillancourt et al. (27), for example, found that peer victimization, depressive symptoms, and elevated salivary cortisol were associated with poorer memory performance in children, suggesting that chronic peer-related stress may affect not only emotional well-being but also neurocognitive functioning. Similarly, Rudolph et al. (28) showed that individual differences in salivary cortisol and salivary alpha-amylase moderated the association between peer victimization and aggression, indicating that both HPA-axis and autonomic nervous system activity may shape adolescents' behavioral responses to social adversity.

Autonomic nervous system functioning is another relevant pathway. Exposure to bullying or relational victimization may be associated with changes in sympathetic arousal and parasympathetic regulation, which are involved in emotional reactivity, behavioral inhibition, and social engagement. Salivary alpha-amylase, often used as an indirect marker of sympathetic nervous system activity, has been examined together with cortisol in studies of peer victimization and aggression (28). In addition, heart rate variability (HRV), commonly considered an index of parasympathetic flexibility and regulatory capacity, may be particularly relevant for understanding why some adolescents exposed to peer victimization develop internalizing symptoms such as anxiety, withdrawal, and depression, whereas others exhibit reactive aggression or behavioral dysregulation. Lower autonomic flexibility may reduce the adolescent's capacity to down-regulate physiological arousal during social conflict, thereby increasing vulnerability to both victimization-related distress and aggressive responses.

Neurobiological models of trauma and social threat further suggest that recurrent victimization may sensitize neural systems involved in threat detection, salience processing, and emotion regulation. Adolescents exposed to chronic peer aggression may develop heightened threat sensitivity, hostile attribution bias, and hypervigilance toward ambiguous social cues. These mechanisms may contribute to different developmental trajectories. In some adolescents, increased threat sensitivity may promote avoidance, social withdrawal, anxiety, and post-traumatic stress symptoms. In others, particularly those with impulsivity or poor inhibitory control, the same heightened sensitivity to threat may increase the likelihood of reactive aggression. Thus, biological stress reactivity may interact with psychological traits such as emotional dysregulation, impulsivity, empathy deficits, and moral disengagement, shaping whether adolescents are more likely to become victims, perpetrators, or bully-victims.

Immune-inflammatory mechanisms may also be relevant. Longitudinal evidence suggests that childhood bullying victimization may be associated with low-grade systemic inflammation later in development. Copeland et al. (29) found that childhood bullying involvement predicted differences in C-reactive protein levels into adulthood, suggesting that peer victimization may have lasting biological consequences. Similarly, Takizawa et al. (30) reported that bullying victimization in childhood predicted inflammation and obesity in mid-life, supporting the hypothesis that early social adversity may become biologically embedded and influence long-term physical and mental health trajectories. These findings are particularly important because inflammation has been implicated in depressive symptoms, fatigue, sleep disturbance, somatic complaints, and stress-related psychopathology.

Research indicates that biological and psychophysiological mechanisms may help explain the heterogeneity of outcomes observed among adolescents exposed to school violence. The same social experience may lead to different consequences depending on the adolescent's stress physiology, neurodevelopmental vulnerabilities, psychiatric profile, family environment, and available social support. Conversely, supportive relationships, positive school climate, trauma-informed interventions, and emotion-regulation training may buffer the biological effects of chronic social stress. Future research should therefore integrate self-report, behavioral, and contextual measures with biological assessments such as diurnal cortisol profiles, cortisol reactivity to social stress, salivary alpha-amylase, HRV, inflammatory biomarkers, sleep parameters, and neurocognitive measures of threat processing. Such integration would strengthen prevention models by identifying adolescents at increased vulnerability and by clarifying how school-based interventions may reduce not only psychological distress but also stress-related biological dysregulation.

### 3.7 Psychiatric vulnerabilities

Several psychiatric conditions are consistently associated with an elevated risk of involvement in bullying and school violence. ADHD, for example, has been repeatedly linked to higher rates of both bullying perpetration and victimization. Core symptoms of ADHD, including impulsivity, emotional dysregulation, and difficulties in social functioning, contribute to increased likelihood of peer conflict and aggressive behavior (8).

Conduct disorder represents another psychiatric condition strongly associated with aggressive behaviors. Youth with conduct disorder often display persistent patterns of rule-breaking, aggression toward others, and violations of social norms, which can mani-fest within school settings as bullying or other forms of violence (31). Mood disorders also influence the emergence of aggressive behaviors among adolescents. Depression has been associated with both heightened vulnerability to victimization and, in some cases, reactive aggression toward peers (4). Emotional instability related to mood dysregulation may further impair the capacity to manage interpersonal conflicts and increase aggressive responses.

These findings underscore the relevance of integrating psychiatric vulnerabilities into models of school violence. Early identification, targeted mental health support, and interventions addressing ADHD, conduct disorder, and mood dysregulation may be critical strategies for reducing aggressive behaviors and improving psychosocial outcomes among adolescents.

## 4 Social and environmental determinants of school violence

While individual psychological vulnerabilities contribute to the emergence of aggressive behaviors, school violence cannot be fully understood without considering the broader social and environmental contexts in which adolescents develop. Ecological and developmental models emphasize that aggressive behaviors arise from dynamic interactions between individual characteristics and contextual influences across multiple levels, including family, peer relationships, school climate, and digital environments (32, 33). These contextual factors may either exacerbate risk or function as protective mechanisms, buffering adolescents from engaging in or experiencing violence. A systematic mapping of the literature indicates that family environment, peer dynamics, school policies, and online interactions collectively shape the likelihood of aggression and victimization.

### 4.1 Family environment and parenting practices

The family environment is a central determinant of children's emotional development, social competencies, and behavioral regulation. Parenting styles characterized by warmth, consistent discipline, and emotional support are consistently associated with lower levels of aggressive behavior among adolescents. Conversely, dysfunctional family environments marked by conflict, neglect, harsh discipline, or inconsistent parenting practices increase the likelihood of aggressive conduct and peer violence (10).

Exposure to domestic violence or family instability can further contribute to the development of maladaptive behavioral patterns. According to social learning theory, children internalize aggressive behaviors by observing and modeling interactions within their family environment (34). Adolescents raised in environments where conflict is frequently resolved through aggression are therefore more likely to reproduce similar behaviors in peer interactions.

Parental monitoring represents a critical protective factor. Empirical evidence shows that adolescents whose parents maintain active supervision of social activities and peer relationships are less likely to engage in bullying behaviors (35). Supportive parent-child relationships strengthen emotional resilience and promote adaptive coping strategies in response to peer conflict.

### 4.2 Peer relationships and social dynamics

Peer relationships represent a critical context influencing adolescent behavior, shaping both risk and protective pathways for school violence. During adolescence, social acceptance and peer status become particularly salient, and peer group dynamics strongly influence behavioral norms within school settings.

Aggressive behaviors may be reinforced within peer groups as a strategy for gaining social dominance or maintaining group status. Research indicates that bullying can function to achieve social power or popularity, especially within competitive social hierarchies (36). In these contexts, aggressive behavior may be tolerated or implicitly rewarded by the peer group.

Conversely, peer relationships can also serve as robust protective factors. Positive friendships, supportive peer networks, and prosocial norms reduce the likelihood of aggressive behavior and promote constructive conflict resolution. Adolescents who feel socially connected and supported by peers are less likely to engage in bullying and more likely to defend victimized classmates (37).

### 4.3 School climate

The broader school environment is a key determinant of student behavior. School climate encompasses the quality of social relationships, norms, and organizational practices within a school. Positive climates, including supportive teacher-student interactions, clear behavioral expectations, and inclusive policies, are associated with lower bullying and aggression (11).

Teachers and school staff actively shape these environments. Educators who monitor student interactions and intervene in bullying establish norms discouraging aggressive behaviors. In contrast, schools where bullying is ignored may inadvertently reinforce aggression.

School connectedness, defined as the degree to which students feel valued and supported within the school community, functions as a protective factor. Strong school belonging promotes positive peer relationships and greater psychological well-being (38).

### 4.4 Community context and socioeconomic disadvantage

School violence is also embedded within the socioeconomic and community contexts surrounding students and educational institutions. Concentrated poverty, residential instability, neighborhood violence, limited access to recreational and mental health resources, and reduced collective efficacy may increase adolescents' cumulative exposure to stress and normalize aggressive responses to interpersonal conflict. Community disadvantage may also constrain schools' capacity to provide adequate supervision, psychological support, extracurricular activities, and stable relationships with families (5, 33).

These associations should not be interpreted deterministically. Most adolescents living in socioeconomically disadvantaged communities do not engage in violence, and structural disadvantage does not constitute an individual behavioral trait. Rather, community conditions may modify exposure to chronic stressors and the availability of protective resources. Community cohesion, safe public spaces, accessible youth services, supportive adult networks, and coordinated school-community partnerships may buffer these risks. Including community context within the biopsychosocial framework therefore shifts part of the explanatory focus from individual deficits to the structural and environmental conditions in which development occurs.

### 4.5 Digital environments and cyberbullying

Rapid expansion of digital technologies has introduced cyberbullying, a form of aggression occurring via social media, messaging applications, and online gaming (12). Unlike traditional bullying, cyberbullying can occur at any time and may reach a broader audience, thereby amplifying the psychological impact of victimization. The perceived anonymity of online environments may also reduce social accountability and increase the likelihood of aggressive behavior.

Research suggests that cyberbullying is associated with psychological outcomes similar to, and sometimes more severe than, those observed in traditional bullying. Victims of cyberbullying frequently report increased levels of depression, anxiety, loneliness, and suicidal ideation (39). Moreover, involvement in cyberbullying often overlaps with traditional forms of bullying, highlighting the interconnected nature of these phenomena.

Prevention strategies must address both online and offline contexts. Educational programs promoting digital literacy, empathy, and responsible online behavior are essential components of comprehensive anti-bullying initiatives.

To provide an integrative overview of the main determinants associated with school violence, Table 1 summarizes the principal individual, relational, and contextual risk and protective factors identified in the literature.

**Table 1:** Multilevel risk and protective factors associated with school violence.

Level	Risk factors	Protective factors	Key evidence
Individual	Emotional dysregulation, impulsivity, empathy deficits, trauma exposure	Emotional regulation skills, social competence	Shields & Cicchetti, 1998; Jolliffe & Farrington, 2004
Psychiatric	ADHD, conduct disorder, mood disorders	Early psychological support	Holmberg & Hjern, 2008; Kim & Leventhal, 2008
Family	Harsh parenting, family conflict, domestic violence	Parental monitoring, supportive parenting	Baldry & Farrington, 2005; Lereya et al., 2013
Peer	Social dominance norms, peer rejection	Prosocial peer relationships, peer support	Salmivalli, 2010
School	Negative school climate, lack of supervision	Positive school climate, teacher involvement	Wang & Degol, 2016
Digital	Cyberbullying, anonymity in online interactions	Digital literacy, online safety education	Kowalski et al., 2014
Biological/psychophysiological	Stress-system dysregulation, altered HPA-axis functioning, heightened autonomic arousal, reduced physiological regulation, increased threat sensitivity	Stress regulation, sleep and emotional recovery, supportive caregiving, trauma-informed interventions	Kliewer et al. 2019; Copeland et al. 2014

ADHD, attention-deficit/hyperactivity disorder; HPA-axis, hypothalamic-pituitary-adrenal axis.

**Figure 6:**
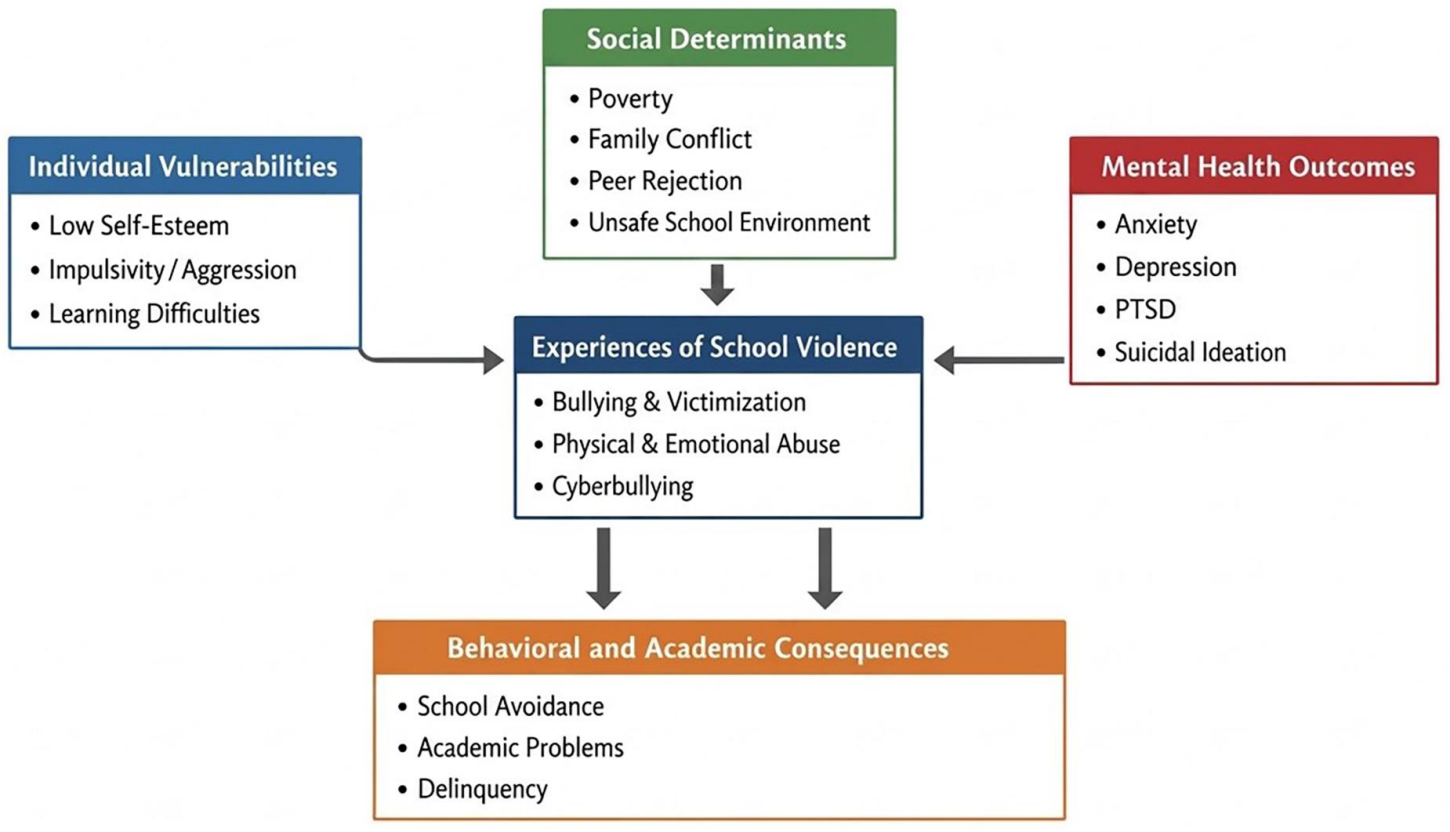
Developmental pathways linking individual vulnerabilities, social determinants, and mental health consequences of school violence. School violence emerges from the interaction between individual psychological vulnerabilities (e.g., emotional dysregulation, impulsivity, psychiatric disorders) and social determinants (family environment, peer relationships, school climate, and digital environments). These interacting factors contribute to bullying and peer victimization dynamics, which in turn may lead to adverse mental health outcomes, including depression, anxiety, trauma-related symptoms, and suicidal ideation. PTSD, post-traumatic stress disorder.

Individual psychological vulnerabilities and broader social determinants interact in shaping the emergence of aggressive behaviors and peer victimization in school environments. From a developmental psychopathology perspective, school violence can be conceptualized as the outcome of multiple interacting risk factors operating across individual, relational, and contextual levels. Figure 6 illustrates a conceptual framework summarizing the main psychological and social pathways contributing to school violence and its mental health consequences.

## 5 Mental health consequences of school violence

Exposure to school violence and bullying represents a significant risk factor for a wide range of adverse mental health outcomes during adolescence and adulthood. A growing body of research indicates that both victims and perpetrators of bullying may experience substantial psychological difficulties, although the nature and severity of these outcomes may vary depending on the individual's role in the bullying dynamic (1). Importantly, adolescents who are both perpetrators and victims, often referred to as bully-victims, tend to exhibit the highest levels of psychological distress and behavioral problems (14).

### 5.1 Depression and anxiety

Depression and anxiety represent two of the most frequently reported mental health consequences associated with exposure to school violence. Numerous longitudinal studies have demonstrated that adolescents who experience peer victimization are at increased risk of developing depressive symptoms, generalized anxiety, and social withdrawal (4, 40–42). Evidence from recent multimodal psychological assessment studies (43–45) highlights novel approaches to detecting depression and anxiety symptoms, which could complement traditional self-report measures in school-aged populations.

The mechanisms linking bullying victimization to internalizing symptoms are com-plex and multifactorial. Repeated experiences of humiliation, rejection, and social exclusion may negatively affect self-esteem and contribute to the development of maladaptive cognitive patterns, including feelings of worthlessness and hopelessness. Over time, these psychological processes may increase vulnerability to depressive disorders (46).

Furthermore, adolescents exposed to chronic peer victimization may develop heightened vigilance and fear of social interactions, contributing to increased levels of social anxiety. Such difficulties may further impair peer relationships and reinforce cycles of isolation and psychological distress.

### 5.2 Post-traumatic stress symptoms

Beyond depression and anxiety, exposure to severe or chronic bullying has increasingly been conceptualized as a potentially traumatic experience. Some victims of persistent peer victimization exhibit symptoms consistent with post-traumatic stress disorder (PTSD), including intrusive memories, hypervigilance, avoidance behaviors, and emotional numbing (47, 48).

The traumatic impact of bullying may be particularly pronounced when victimization occurs repeatedly over time or involves multiple forms of aggression, such as physical intimidation, social exclusion, and online harassment (49, 50). For adolescents who experience bullying in multiple contexts, both offline and online, the cumulative psychological burden may significantly increase the risk of trauma-related symptoms.

### 5.3 Suicidal ideation and self-harm

One of the most concerning consequences of school violence is its association with suicidal ideation and self-harm behaviors among adolescents. Research has consistently demonstrated that involvement in bullying, particularly as a victim, is associated with increased risk of suicidal thoughts and suicide attempts (51, 52).

Victimization may contribute to suicidal ideation through several pathways, including the development of depressive symptoms, social isolation, and feelings of helplessness. Some studies have shown that both perpetrators and victims of bullying may experience elevated suicide risk, suggesting that aggressive behavior itself may reflect underlying psychological distress (53).

Given the severity of these outcomes, early identification of bullying involvement represents an important component of suicide prevention strategies within school settings.

### 5.4 Self-esteem and substance-related outcomes

Exposure to repeated bullying may affect adolescents' developing self-concept. Persistent devaluation by peers, public humiliation, exclusion, and perceived loss of social status can reinforce negative beliefs about personal worth, competence, and social acceptability. Reduced self-esteem may subsequently mediate part of the association between victimization and depression, anxiety, social withdrawal, and hopelessness. Because self-esteem is shaped by reciprocal interactions between the individual and the social environment, longitudinal designs are required to distinguish pre-existing vulnerability from changes attributable to victimization (46, 48).

Bullying involvement has also been associated with substance-related risk behaviors. Victims may use alcohol or other substances to cope with negative affect or facilitate social belonging, whereas perpetrators and bully-victims may show substance use within a broader pattern of disinhibition, rule-breaking, and affiliation with deviant peers. Bully-victims may be especially vulnerable because they combine victimization-related distress with externalizing and behavioral-control difficulties. Screening adolescents involved in school violence should therefore include age-appropriate assessment of alcohol, nicotine, cannabis, and other substance use, while avoiding assumptions that all affected students follow the same developmental pathway.

### 5.5 Academic and social consequences

School violence may also negatively affect adolescents' academic performance and social development. Students who experience bullying frequently report difficulties concentrating in class, reduced academic motivation, and increased school absenteeism (54). In some cases, persistent victimization may lead to school avoidance or dropout. Evidence mapping in the scoping review highlights that both perpetrators and bully-victims can experience academic impairments, although mechanisms may differ.

In addition to academic consequences, bullying can significantly disrupt adolescents' social development. Peer victimization may lead to social withdrawal, mistrust of peers, and difficulties forming supportive relationships. Over time, these experiences may inter-fere with the development of essential social competencies, including communication skills, empathy, and cooperative behaviors (55).

Moreover, the social stigma associated with bullying victimization may reinforce marginalization within peer groups, further exacerbating psychological distress and limiting opportunities for positive social engagement.

Research underscores the profound and multifaceted impact of school violence on adolescents' mental health and developmental trajectories. Recognizing these consequences highlights the importance of early intervention and comprehensive prevention strategies aimed at reducing exposure to violence in school environments. To synthesize the principal psychological consequences associated with school violence, Table 2 summarizes the main mental health outcomes identified in the literature among adolescents involved in bullying dynamics.

**Table 2:** Major mental health outcomes associated with school violence and bullying involvement.

Outcome	Description	Evidence from literature
Depression	Victims of bullying show higher levels of depressive symptoms and emotional distress	Copeland et al., 2013; Arseneault, 2018
Anxiety	Peer victimization is associated with social anxiety, fear of social situations, and emotional insecurity	Reijntjes et al., 2010
Post-traumatic stress symptoms	Chronic or severe bullying may produce trauma-related symptoms including hypervigilance and intrusive memories	Idsoe et al., 2012
Suicidal ideation and self-harm	Both victims and perpetrators show increased risk of suicidal thoughts and behaviors	Holt et al., 2015; Klomek et al., 2010
Academic difficulties	Victimization is associated with reduced concentration, lower academic performance, and school absenteeism	Nakamoto & Schwartz, 2010
Social withdrawal	Victims may develop social isolation and difficulties in peer relationships	Arseneault, 2018

## 6 Prevention and intervention strategies in school violence

Preventing school violence requires comprehensive and developmentally informed approaches that address both individual vulnerabilities and contextual risk factors. Research increasingly indicates that the most effective interventions are multilevel strategies targeting students, families, schools, and broader social environments. Within a develop-mental psychopathology framework, prevention efforts should focus not only on reducing aggressive behaviors but also on promoting emotional regulation, social competence, and supportive educational climates (33).

### 6.1 School-based prevention programs

School-based programs represent one of the most widely studied and effective approaches for preventing bullying and school violence. These interventions typically adopt a whole-school strategy aimed at modifying social norms, strengthening teacher engagement, and promoting positive peer interactions (56).

Evidence suggests that programs addressing multiple components, such as classroom activities, school policies, and teacher training, are more effective than isolated interventions targeting individual students (57).

One of the most extensively researched models is the Olweus Bullying Prevention Program, which focuses on improving school climate, increasing adult supervision, and establishing clear behavioral rules against bullying (58). Evaluations of this program have demonstrated significant reductions in bullying behaviors and improvements in peer relationships across multiple countries.

Another widely implemented intervention is the KiVa program, developed in Finland. This program emphasizes the role of bystanders in bullying situations and aims to alter group dynamics that reinforce aggressive behaviors. Studies evaluating KiVa have shown substantial reductions in bullying victimization and perpetration, as well as improvements in students' empathy and prosocial attitudes.

In addition to anti-bullying programs specifically designed to reduce aggression, broader social-emotional learning (SEL) initiatives have demonstrated significant benefits in promoting emotional regulation, empathy, and interpersonal problem-solving skills (59). Meta-analytic evidence indicates that SEL programs can improve students' social competencies, reduce behavioral problems, and enhance academic outcomes (60). By strengthening emotional and interpersonal skills, these interventions may reduce the likelihood that adolescents engage in aggressive or violent behaviors within school settings.

### 6.2 Family-based interventions

Family environments play a central role in shaping children's emotional development and behavioral regulation. Consequently, prevention strategies targeting parenting practices and family functioning represent an important component of comprehensive anti-violence initiatives.

Research has consistently shown that supportive parenting characterized by warmth, consistent discipline, and active monitoring of adolescents’ social activities is associated with lower levels of aggressive behavior and bullying involvement (35). Parenting interventions aimed at improving communication, emotional responsiveness, and conflict resolution skills can therefore contribute to reducing the risk of school violence.

Programs that involve parents in school-based prevention efforts may be particularly effective. Collaborative initiatives between schools and families can enhance parental awareness of bullying dynamics and encourage early intervention when behavioral difficulties emerge. In addition, family-focused therapeutic approaches may help address underlying psychological vulnerabilities, such as emotional dysregulation or trauma expo-sure, that contribute to aggressive behaviors among adolescents.

### 6.3 Mental health screening and early identification

Early identification of psychological vulnerabilities represents another key strategy for preventing school violence. Many adolescents involved in bullying dynamics exhibit underlying mental health difficulties, including emotional dysregulation, impulsivity, trauma-related symptoms, or psychiatric disorders such as attention-deficit/hyperactivity disorder and conduct disorder.

School-based mental health screening programs can help identify students at risk of behavioral difficulties and provide timely access to appropriate psychological support. Integrating mental health professionals within school settings may facilitate early detection of emotional and behavioral problems and improve coordination between educational and clinical services (61).

Moreover, interventions targeting emotional regulation and coping skills have shown promise in reducing aggressive behaviors among adolescents. Cognitive-behavioral approaches, mindfulness-based interventions, and trauma-informed programs may help students develop more adaptive strategies for managing stress, anger, and interpersonal conflicts.

Given the strong association between bullying involvement and suicidal ideation, early mental health assessment is also essential for suicide prevention efforts within school environments (51). Students who experience chronic victimization may require targeted psychological interventions aimed at addressing depressive symptoms, social withdrawal, and trauma-related distress.

### 6.4 Digital prevention strategies and cyberbullying

The increasing prevalence of cyberbullying highlights the need for prevention strategies that address digital environments alongside traditional school contexts. Online harassment can occur continuously and may reach large audiences, thereby intensifying the psychological impact of victimization.

Educational initiatives promoting digital literacy, empathy, and responsible online behavior represent important components of contemporary anti-bullying strategies. Programs that teach adolescents how to navigate online interactions safely and ethically may help reduce aggressive behaviors within digital platforms (39).

In addition, schools can implement policies aimed at monitoring online harassment and providing clear procedures for reporting cyberbullying incidents. Collaboration between educators, parents, and technology platforms may further enhance prevention efforts by promoting safer digital environments for adolescents.

**Figure 7:**
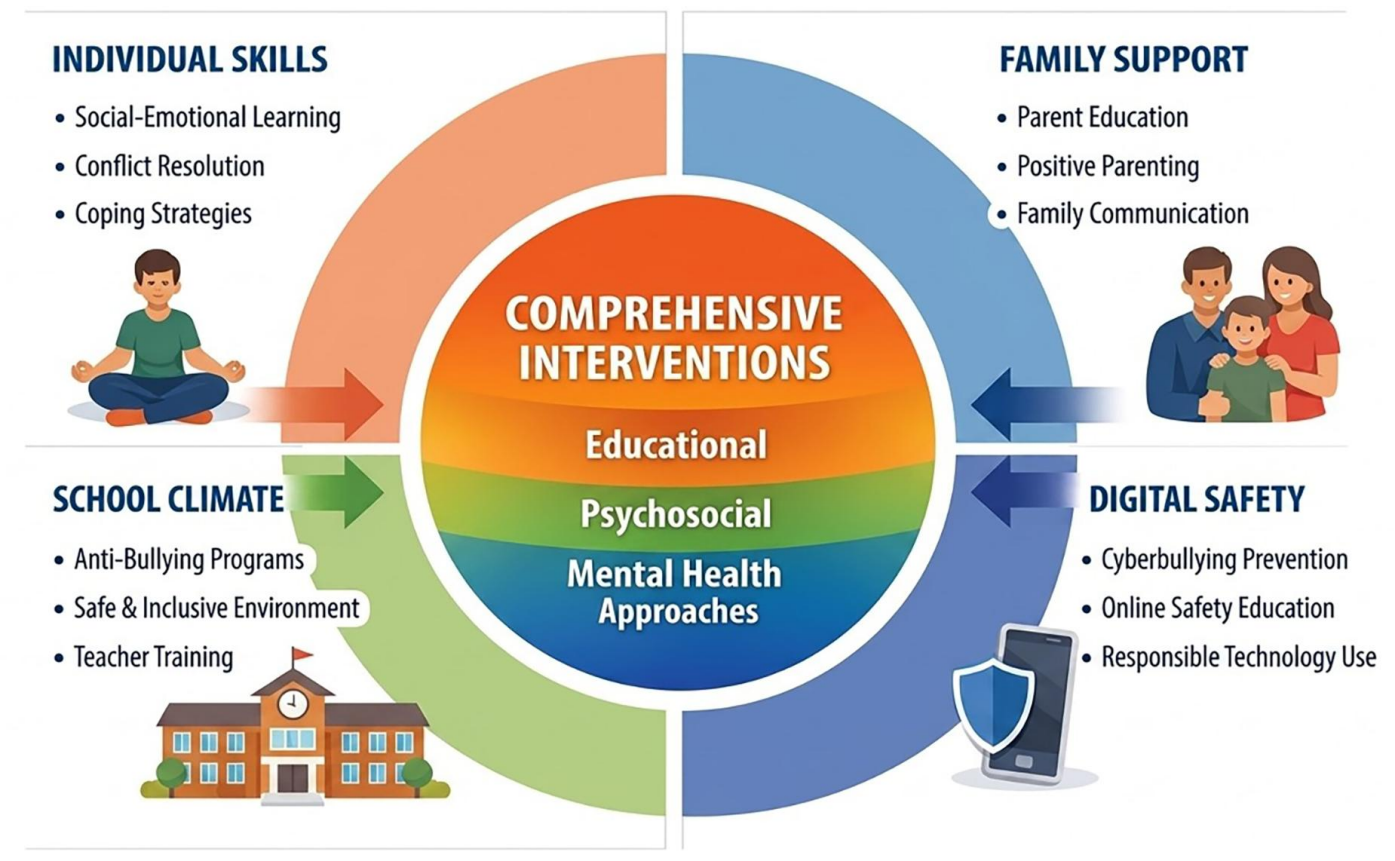
Multilevel prevention framework for school violence. Prevention strategies targeting individual psychological skills, family dynamics, school cli-mate, and digital environments may contribute to reducing bullying behaviors and improving adolescent mental health outcomes. Comprehensive interventions integrating educational, psychosocial, and mental health approaches are likely to produce the most effective and sustainable reductions in school violence.

Overall, prevention strategies that integrate school-based programs, family involvement, mental health support, and digital education are more likely to produce sustainable reductions in school violence. Such comprehensive approaches reflect the complex and multifactorial nature of bullying behaviors and align with developmental models emphasizing the interaction between individual vulnerabilities and social environments. Figure 7 illustrates a multilevel prevention framework summarizing the main intervention domains that may reduce school violence and improve adolescent mental health outcomes.

### 6.5 Biologically informed prevention: targeting stress regulation and arousal

A biologically informed approach to prevention should not be limited to identifying psychosocial risk factors, but should also consider how interventions may reduce chronic stress activation and improve physiological regulation. School-based anti-bullying programs, socialemotional learning, trauma-informed education, mindfulness-based strategies, sleep promotion, physical activity, and family support may contribute to reducing allostatic load by improving emotion regulation, decreasing perceived threat, strengthening social safety, and enhancing autonomic flexibility. Although most prevention trials have not included biological endpoints, future intervention studies should consider incorporating measures such as diurnal cortisol, cortisol reactivity, heart rate variability, sleep parameters, and inflammatory biomarkers. This would allow researchers to deter-mine whether effective school-based interventions reduce not only bullying behaviors and psychological symptoms, but also stress-related biological dysregulation.

## 7 Discussion

The present scoping review examined the psychological and social pathways contributing to violence in school environments from a developmental psychopathology perspective. It maps the existing literature to identify key risk factors, social determinants, mental health outcomes, and prevention strategies, highlighting areas where evidence is robust or limited. It integrates evidence from developmental psychopathology, educational psychology, and mental health research to provide a comprehensive framework for under-standing school violence.

A central implication of this review is that school violence should be understood not only as an educational, behavioral, or psychosocial problem, but also as a repeated social-stress exposure with potential neurobiological consequences. During adolescence, the systems involved in stress regulation, emotional control, reward processing, social cognition, and threat detection are still undergoing maturation. Recurrent exposure to peer aggression may therefore interfere with developmental processes by repeatedly activating the HPA axis and autonomic nervous system. Over time, this may contribute to maladaptive patterns of stress reactivity, including either hyperactivation or blunting of cortisol responses, sympathetic overarousal, reduced parasympathetic flexibility, and increased vigilance toward social threat. These mechanisms may help explain why some victimized adolescents develop anxiety, depression, social withdrawal, sleep problems, or post-traumatic stress symptoms, whereas others show irritability, reactive aggression, impulsivity, or conduct problems. In this sense, biological stress reactivity may function both as a mediator of the effects of school violence on mental health and as a moderator of individual vulnerability or resilience.

The available evidence suggests that school violence emerges from the com-plex interaction between individual vulnerabilities, relational dynamics, and broader environmental contexts. Rather than representing an isolated behavioral phenomenon, aggressive behaviors in school settings appear to reflect multidimensional developmental processes shaped by emotional, cognitive, and social factors operating across different ecological levels.

One of the central findings emerging from the literature is the critical role of individual psychological vulnerabilities in the development of aggressive behavior among adolescents. Emotional dysregulation, impulsivity, and deficits in empathy have consistently been associated with increased risk of bullying perpetration and peer conflict. These psychological characteristics may reduce adolescents' capacity to regulate emotional responses and manage interpersonal disagreements in adaptive ways. From a developmental psychopathology perspective, difficulties in emotional regulation may reflect disruptions in early developmental processes involving attachment relationships, emotion socialization, and stress regulation systems (5). Consequently, aggressive behaviors may in part represent maladaptive coping strategies used by adolescents who struggle to manage intense emotional states.

Existing literature also highlights the significant role of trauma exposure and adverse childhood experiences. Exposure to abuse, neglect, or household dysfunction has been associated with alterations in stress response systems, increased emotional reactivity, and difficulties in interpersonal functioning (26). These developmental disruptions may increase the likelihood of both aggressive behavior and peer victimization. Adolescents who have experienced early adversity may display heightened threat sensitivity and mistrust in social interactions, potentially contributing to cycles of aggression and retaliation within peer relationships.

A further contribution of this review is the integration of biological and psychophysiological mechanisms within the broader developmental model of school violence. Although literature remains heterogeneous, the available evidence suggests that peer victimization and chronic exposure to school-based threat may influence stress-response systems, autonomic regulation, and neurodevelopmental processes involved in emotional reactivity and behavioral control. These mechanisms may partly explain why some adolescents exposed to bullying develop internalizing symptoms, such as depression, anxiety, and post-traumatic stress symptoms, whereas others show externalizing behaviors, including reactive aggression and conduct problems. Importantly, these biological mechanisms operate in continuous interaction with psychological and social determinants, supporting the need for a biopsychosocial rather than purely behavioral understanding of school violence.

It is important to stress that school violence cannot be fully understood without considering the broader social environment in which adolescents develop. Family dynamics, peer relationships, school climate, and digital environments all contribute to shaping behavioral norms and social interactions within school settings. Supportive parenting practices characterized by warmth, emotional availability, and consistent discipline appear to function as protective factors against aggressive behavior, whereas harsh or inconsistent parenting practices may increase the likelihood of involvement in bullying dynamics (35). Similarly, peer group dynamics may either reinforce aggressive behavior or promote pro-social interactions depending on prevailing social norms within the group (62).

The role of school climate represents another crucial contextual factor influencing the prevalence of school violence. Educational environments characterized by supportive teacher-student relationships, clear behavioral expectations, and effective disciplinary policies tend to exhibit lower rates of bullying and aggression (11). Teachers and school staff play an essential role in establishing social norms that discourage violence and promote respectful interactions among students. When bullying behaviors are consistently addressed and monitored by school personnel, students may perceive aggressive behavior as less socially acceptable, thereby reducing its occurrence.

In recent years, the expansion of digital communication technologies has introduced new forms of peer aggression that extend beyond traditional school boundaries. Cyber-bullying has become an increasingly prominent concern due to its pervasive nature and potential to expose victims to continuous harassment. Unlike traditional bullying, cyber-bullying may occur at any time and can reach large audiences, thereby amplifying its psychological impact (12). The overlap between online and offline forms of victimization further highlights the need to address digital environments within comprehensive prevention strategies.

Another important aspect highlighted by the present review concerns the substantial mental health consequences associated with exposure to school violence. Adolescents involved in bullying dynamics, particularly those who experience chronic victimization, demonstrate increased vulnerability to a range of psychological difficulties including de-pression, anxiety, trauma-related symptoms, and suicidal ideation (1, 51). These findings underscore the importance of recognizing school violence not only as a behavioral or disciplinary issue but also as a significant public health concern with long-term implications for adolescent mental health.

The evidence reviewed in this article also supports the importance of integrated prevention strategies that address multiple levels of influence simultaneously. School-based programs targeting social norms, emotional regulation skills, and peer relationships have demonstrated promising results in reducing bullying behaviors and improving school climate. Interventions such as the Olweus Bullying Prevention Program and the KiVa program illustrate how comprehensive approaches involving students, teachers, and school administrators can significantly reduce bullying prevalence (58, 63). Furthermore, social-emotional learning programs may enhance adolescents' ability to manage emotions, empathize with others, and resolve interpersonal conflicts constructively (60).

At the same time, prevention efforts should not be limited to school settings alone. Family-based interventions and parental involvement represent important components of comprehensive prevention strategies. Strengthening parent-child relationships and promoting supportive parenting practices may enhance adolescents' emotional resilience and reduce the likelihood of aggressive behavior. Additionally, integrating mental health services within school environments may facilitate early identification of psychological vulnerabilities and provide timely support to students experiencing emotional or behavioral difficulties.

To integrate these findings within a biopsychosocial framework, Table 3 summarizes the principal biological, psychological, social, and digital mechanisms through which school violence may influence adolescent mental health outcomes. This synthesis highlights how peer victimization and aggressive behaviors are not explained by a single pathway, but rather emerge from the interaction between stress-related biological processes, individual psychological vulnerabilities, relational dynamics, school climate, and digital exposure.

**Table 3:** Biopsychosocial mechanisms linking school violence to adolescent mental health outcomes.

Domain	Mechanism	Possible consequence	Examples of measures
Biological/neuroendocrine	HPA-axis dysregulation, altered cortisol secretion, atypical stress reactivity	Anxiety, depression, trauma-related symptoms, sleep disruption	Diurnal cortisol, cortisol awakening response, cortisol reactivity
Biological/autonomic	Sympathetic arousal, reduced parasympathetic flexibility, reduced HRV	Hypervigilance, emotional dysregulation, reactive aggression, social withdrawal	HRV, heart rate, salivary alpha-amylase
Biological/inflammatory	Low-grade systemic inflammation	Depressive symptoms, fatigue, somatic complaints, long-term health vulnerability	CRP, IL-6, fibrinogen
Psychological	Emotional dysregulation, impulsivity, empathy deficits, moral disengagement	Bullying perpetration, victimization, bully-victim profile	Emotion regulation scales, impulsivity scales, empathy measures
Trauma-related	Threat sensitivity, hypervigilance, hostile attribution bias	PTSD symptoms, avoidance, reactive aggression, interpersonal mistrust	Trauma questionnaires, PTSD symptom scales, threat-processing tasks
Family/relational	Harsh parenting, family conflict, poor parental monitoring, low support	Aggression, victimization, poor coping, persistence of bullying dynamics	Family functioning scales, parental monitoring measures
Peer/school	Peer rejection, social dominance norms, negative school climate, poor supervision	Peer conflict, normalization of bullying, academic difficulties	Peer nomination, school climate scales, teacher reports
Digital	Cyberbullying, online disinhibition, persistent digital exposure	Anxiety, depression, suicidal ideation, social withdrawal	Cyberbullying questionnaires, digital-use assessment

The mechanisms listed in the table are not mutually exclusive and may interact dynamically across development. HPA, hypothalamic–pituitary–adrenal; HRV, heart rate variability; CRP, C-reactive protein; IL-6, interleukin-6; PTSD, post-traumatic stress disorder.

Despite the growing body of research on school violence, several limitations in the existing literature should be acknowledged. Many studies rely on cross-sectional designs, which limit the ability to establish causal relationships between risk factors and aggressive behaviors. Longitudinal research is needed to better understand the developmental trajectories that lead to bullying involvement over time. Variations in definitions and measurement of bullying across studies may contribute to inconsistencies in prevalence estimates and risk factor identification. Cultural and contextual differences across educational systems may influence the manifestation and interpretation of aggressive behaviors, highlighting the need for cross-cultural research in this field. Another limitation concerns the relatively limited integration of biological and psychophysiological measures in the existing literature. Most studies rely on self-report or behavioral assessments, while fewer studies include objective markers such as cortisol, HRV, inflammatory biomarkers, sleep parameters, or neurocognitive measures of threat processing. This limits the possibility of clarifying the biological pathways through which peer victimization and school violence may become embedded in adolescent development.

This review was not prospectively registered in an international registry, and a publicly accessible protocol was not published before study selection. At the time the review was initiated, the authors developed an internal review plan but did not deposit it in an external repository. Although prospective registration is not uniformly mandatory for scoping reviews and some registries do not routinely accept this review type, protocol publication or deposition in an open repository would have increased methodological transparency, reduced the possibility of *post hoc* decisions, and allowed clearer comparison between planned and completed methods. This should therefore be considered a limitation of the present review.

Future research should aim to further clarify the developmental mechanisms linking early psychological vulnerabilities, environmental stressors, and aggressive behaviors in adolescence. Longitudinal studies examining the interaction between emotional regulation, social relationships, and digital environments may provide valuable insights into the emergence and persistence of school violence. In addition, evaluating the long-term effectiveness of prevention programs across different cultural contexts represents an important direction for future investigation. Future studies should also adopt multimodal and longitudinal designs integrating psychosocial assessments with biological and psychophysiological measures, including cortisol profiles, HRV, inflammatory biomarkers, sleep in-dicators, and neurocognitive indices of threat processing. Such approaches may clarify mechanisms of vulnerability and resilience and inform early identification of adolescents at highest risk.

Overall, understanding school violence through the lens of developmental psychopathology allows for a more comprehensive conceptualization of the phenomenon. By integrating psychological, social, and environmental perspectives, researchers and practitioners may develop more effective strategies for preventing violence in school environments and promoting healthier developmental trajectories for adolescents. For child and adolescent psychiatry, findings of existing research highlight the importance of routinely assessing bullying involvement, peer victimization, cyberbullying, trauma exposure, emotional dysregulation, and suicidal ideation in clinical evaluations. School violence may represent both a risk factor for psychopathology and a clinical marker of broader developmental vulnerability requiring integrated school, family, and mental health interventions.

## 8 Conclusions

School violence represents a complex developmental phenomenon shaped by the interaction of psychological vulnerabilities and broader social contexts. The evidence reviewed in this scoping review suggests that aggressive behaviors in school environments cannot be fully understood as isolated disciplinary problems but rather as outcomes of multifactorial processes involving emotional regulation difficulties, impulsivity, trauma exposure, and psychiatric vulnerabilities interacting with family dynamics, peer relationships, school climate, and digital environments.

From a developmental psychopathology perspective, bullying and other forms of school violence reflect dynamic pathways that unfold across adolescence and are influenced by both risk and protective factors operating at multiple ecological levels. The scoping evidence highlights that these pathways are documented across diverse populations and contexts, although research gaps remain regarding intervention effectiveness and long-term outcomes. Importantly, involvement in bullying, whether as perpetrators, victims, or bully-victims, is associated with significant mental health consequences, including depression, anxiety, trauma-related symptoms, and suicidal ideation, underscoring the relevance of school violence as a major public health concern.

Effective prevention therefore requires comprehensive and multilevel strategies integrating school-based programs, family involvement, mental health screening, and interventions addressing digital environments. Educational institutions, families, and mental health professionals must collaborate to promote supportive school climates, strengthen emotional and social competencies, and ensure early identification of psychological vulnerabilities among students.

Advancing research in this field will require longitudinal studies and interdisciplinary approaches capable of clarifying the developmental mechanisms underlying school violence and informing more effective prevention and intervention strategies. Future work should continue to map emerging trends in cyberbullying, novel risk factors, and intervention outcomes to guide evidence-informed policy and practice. Besides, it should move beyond exclusively psychosocial models by incorporating biological and psychophysiological measures, including cortisol patterns, autonomic functioning, heart rate variability, inflammatory markers, and stress-reactivity paradigms. Such integration may help identify adolescents who are particularly vulnerable to the mental health consequences of school violence and inform more personalized prevention and intervention strategies.
