# Supplementary material for: Biopsychosocial risk pathways to school violence in youth: a scoping review of stress reactivity, mental health outcomes, and prevention
Source: Front Child Adolesc Psychiatry. 2026 Jul 14;5:1895435. doi: 10.3389/frcha.2026.1895435 (PMC13408263; doi:10.3389/frcha.2026.1895435)
Supplement: Supplementary file 1 [file Table1.docx]

**Supplementary Table S1.** Database-specific search strategies and study-identification results.

| Source | Complete search equation | Limits | Search period/date | Records identified |
| --- | --- | --- | --- | --- |
| PubMed/MEDLINE | (("school violence"[Title/Abstract] OR bullying[Title/Abstract] OR "peer victimization"[Title/Abstract] OR "peer aggression"[Title/Abstract] OR "adolescent aggression"[Title/Abstract] OR cyberbullying[Title/Abstract]) AND (child*[Title/Abstract] OR adolescen*[Title/Abstract] OR youth[Title/Abstract] OR student*[Title/Abstract]) AND ("emotional dysregulation"[Title/Abstract] OR impulsiv*[Title/Abstract] OR empathy[Title/Abstract] OR "moral disengagement"[Title/Abstract] OR trauma[Title/Abstract] OR "adverse childhood experiences"[Title/Abstract] OR ADHD[Title/Abstract] OR "attention-deficit/hyperactivity disorder"[Title/Abstract] OR "conduct disorder"[Title/Abstract] OR depression[Title/Abstract] OR anxiety[Title/Abstract] OR suicid*[Title/Abstract] OR "self-harm"[Title/Abstract] OR "self-esteem"[Title/Abstract] OR "substance use"[Title/Abstract] OR family[Title/Abstract] OR parenting[Title/Abstract] OR peer*[Title/Abstract] OR "school climate"[Title/Abstract] OR "school connectedness"[Title/Abstract] OR communit*[Title/Abstract] OR disadvantage*[Title/Abstract] OR prevention[Title/Abstract] OR intervention[Title/Abstract] OR "stress reactivity"[Title/Abstract] OR "HPA axis"[Title/Abstract] OR "hypothalamic-pituitary-adrenal axis"[Title/Abstract] OR cortisol[Title/Abstract] OR autonomic[Title/Abstract] OR "heart rate variability"[Title/Abstract] OR "physiological arousal"[Title/Abstract] OR neuroendocrine[Title/Abstract] OR inflammation[Title/Abstract] OR "allostatic load"[Title/Abstract] OR "threat sensitivity"[Title/Abstract] OR neurodevelopment*[Title/Abstract])) | English language; publications from January 2000 to January 2025 | Final search conducted in January 2025 | NR* |
| Scopus | TITLE-ABS-KEY ("school violence" OR bullying OR "peer victimization" OR "peer aggression" OR "adolescent aggression" OR cyberbullying) AND TITLE-ABS-KEY (child* OR adolescen* OR youth OR student*) AND TITLE-ABS-KEY ("emotional dysregulation" OR impulsiv* OR empathy OR "moral disengagement" OR trauma OR "adverse childhood experiences" OR ADHD OR "attention-deficit/hyperactivity disorder" OR "conduct disorder" OR depression OR anxiety OR suicid* OR "self-harm" OR "self-esteem" OR "substance use" OR family OR parenting OR peer* OR "school climate" OR "school connectedness" OR communit* OR disadvantage* OR prevention OR intervention OR "stress reactivity" OR "HPA axis" OR "hypothalamic-pituitary-adrenal axis" OR cortisol OR autonomic OR "heart rate variability" OR "physiological arousal" OR neuroendocrine OR inflammation OR "allostatic load" OR "threat sensitivity" OR neurodevelopment*) AND PUBYEAR > 1999 AND PUBYEAR < 2026 | English language; publications from January 2000 to January 2025 | Final search conducted in January 2025 | NR* |
| Web of Science Core Collection | TS=("school violence" OR bullying OR "peer victimization" OR "peer aggression" OR "adolescent aggression" OR cyberbullying) AND TS=(child* OR adolescen* OR youth OR student*) AND TS=("emotional dysregulation" OR impulsiv* OR empathy OR "moral disengagement" OR trauma OR "adverse childhood experiences" OR ADHD OR "attention-deficit/hyperactivity disorder" OR "conduct disorder" OR depression OR anxiety OR suicid* OR "self-harm" OR "self-esteem" OR "substance use" OR family OR parenting OR peer* OR "school climate" OR "school connectedness" OR communit* OR disadvantage* OR prevention OR intervention OR "stress reactivity" OR "HPA axis" OR "hypothalamic-pituitary-adrenal axis" OR cortisol OR autonomic OR "heart rate variability" OR "physiological arousal" OR neuroendocrine OR inflammation OR "allostatic load" OR "threat sensitivity" OR neurodevelopment*) | English language; publications from January 2000 to January 2025 | Final search conducted in January 2025 | NR* |
| Total records identified through electronic databases | Combined results from PubMed/MEDLINE, Scopus, and Web of Science Core Collection | Same limits as above | January 2025 | 1,248 |
| Manual reference-list searching | Reference lists of eligible studies and relevant reviews were screened for additional potentially eligible publications | Same eligibility criteria as the database search | January 2025 | 37 |
| Total records identified before duplicate removal | Electronic databases plus manual searching | — | — | 1,285 |
| Duplicates removed | Duplicate records identified across databases and manual sources | — | — | 312 |
| Records remaining for title and abstract screening | Total identified records minus duplicates | — | — | 973 |

*Database-specific retrieval counts were not retained separately. The combined electronic database search identified 1,248 records before duplicate removal.

Abbreviations: ADHD, attention-deficit/hyperactivity disorder; HPA, hypothalamic-pituitary-adrenal; NR, not retained; TITLE-ABS-KEY, title, abstract, and keywords; TS, topic search.
