# Supplementary material for: Biopsychosocial risk pathways to school violence in youth: a scoping review of stress reactivity, mental health outcomes, and prevention
Source: Front Child Adolesc Psychiatry. 2026 Jul 14;5:1895435. doi: 10.3389/frcha.2026.1895435 (PMC13408263; doi:10.3389/frcha.2026.1895435)
Supplement: Supplementary file 2 [file Table2.docx]

**Supplementary Table S2.** Standardized data-charting matrix used for the qualitative evidence mapping.

| Data-charting domain | Information recorded |
| --- | --- |
| Bibliographic information | First author, publication year, country |
| Study characteristics | Study design, recruitment method, data-collection method |
| Population | Sample size, age range or mean age, sex/gender distribution, school level |
| Educational setting | Primary school, secondary school, high school, or other educational context |
| Violence or bullying exposure | School violence, physical bullying, verbal bullying, relational aggression, peer victimization, cyberbullying |
| Participant role | Perpetrator, victim, bully-victim, bystander, or mixed population |
| Psychological factors | Emotional dysregulation, impulsivity, empathy, moral disengagement, self-esteem, coping, trauma exposure |
| Psychiatric factors | Attention-deficit/hyperactivity disorder, conduct problems, mood symptoms, anxiety, post-traumatic stress symptoms, substance use |
| Biological and psychophysiological factors | Hypothalamic-pituitary-adrenal-axis functioning, cortisol, autonomic arousal, heart rate variability, inflammatory markers, stress reactivity, threat processing |
| Family and peer factors | Parenting, family functioning, domestic conflict, peer support, peer norms, social status |
| School and community factors | School climate, teacher support, school connectedness, socioeconomic disadvantage, neighborhood violence, community resources |
| Digital factors | Cyberbullying, social media exposure, online harassment, digital literacy |
| Outcomes | Internalizing symptoms, externalizing symptoms, suicidal ideation, self-harm, academic performance, absenteeism, social functioning |
| Prevention or intervention characteristics | Program type, target population, setting, intervention components, duration, reported outcomes |
| Evidence-mapping category | Biological, psychological, relational, school, community, digital, outcome, or prevention domain |
| Main findings | Principal findings relevant to the review questions |
